# Significant Disparities Exist in Consumer Health Insurance Literacy: Implications for Health Care Reform

**DOI:** 10.3928/24748307-20190923-01

**Published:** 2019-11-05

**Authors:** Jean Edward, Amanda Wiggins, Malea Hoepf Young, Mary Kay Rayens

## Abstract

**Background:**

Health insurance literacy (HIL) is defined as a person's ability to seek, obtain, and understand health insurance plans, and once enrolled use their insurance to seek appropriate health care services.

**Objective:**

The objectives of this study were to assess sociodemographic disparities in HIL, including knowledge of health insurance terms and costs, and confidence in using insurance to access health care in a nationally representative adult sample.

**Methods:**

We conducted a secondary data analysis of the Health Reform Monitoring Survey, which included 15,168 adults age 18 years and older who responded to surveys in the third quarter of 2015 and first quarter of 2016. Rao-Scott chi-square tests and weighted logistic regression were used for analysis.

**Key Results:**

The majority of our sample (51%) reported having inadequate HIL as measured by knowledge of basic insurance terms, and close to one-half (48%) had low confidence in using their insurance to access health care. Logistic regression analysis indicated significant disparities in HIL, with multiple groups identified as being at higher risk for having inadequate HIL (as measured by both knowledge and use of health insurance). These included young adults, women, those with Hispanic ethnicity, those who were not U.S. citizens, and those who were currently unmarried. Also identified to be at risk were those who are unemployed, uninsured, and enrolled in public health insurance plans, and those with lower levels of education and income. Most had inadequate knowledge of their annual out-of-pocket costs and insurance plan's deductible amounts.

**Conclusions:**

One-half of U.S. adults rate themselves as having inadequate HIL. Sociodemographic disparities in self-reported HIL underscore the need for increased consumer education, as well as efforts to simplify the health care system by promoting value-based care, supporting delivery system reforms, and designing services to be responsive to consumer HIL needs and abilities. **[*HLRP: Health Literacy Research and Practice*. 2019;3(4):e250–e258.]**

**Plain Language Summary:**

In a nationally representative sample of 15,168 adults, the majority had low knowledge about basic health insurance terms and had difficulty using health insurance to access needed health care services. These findings indicate that health insurance literacy is a major concern in our community that disproportionately affects some underserved groups more than others, including young adults, groups with low-income, and people who are uninsured.

Health literacy is a critical need in the United States, with research consistently demonstrating that more than one-half of the U.S. population does not understand basic health insurance terms (American Institutes of CPAs, 2013; Kenny, Karpman & Long, 2013; Paez et al., 2014; Quincy, 2012). Despite this limitation, health reform policies continue to place increasing responsibility on consumers to independently make critical, informed decisions about their health insurance and health care needs (Long et al., 2014). Health insurance literacy (HIL) is defined as a person's ability to seek, obtain, and understand health insurance plans, and once enrolled use their insurance to seek appropriate health care services (Quincy, 2012). Without adequate levels of HIL, consumers are unable to understand the financial and health implications of insurance plans, often leading to delayed care or foregoing needed care (Brown et al., 2016; Kim, Braun & Williams, 2013).

Examining HIL helps us better understand barriers to health care access, as well as the financial and quality trends in a competitive health care market (Barnes, Hanoch & Rice, 2015). Improving HIL is essential to supporting consumer health insurance decision-making by increasing their likelihood of being insured. Having health care coverage results in timely access to needed health care services that are vital for disease prevention and management (Hadley, 2007; Durham et al., 1998; Wilper et al., 2009a; Wilper et al., 2009b). Despite growing research in the area of HIL after the implementation of the Affordable Care Act (ACA) and subsequent changes in health care reform policies, few studies have been able to establish national sociodemographic trends in HIL (Bartholomae, Russell, Braun, & McCoy, 2016; Brown et al., 2016; Harris-Kojetin et al., 2007; Kullgren et al., 2010; Politi et al., 2014; Sinaiko, Ross-Degnan, Soumerai, Lieu, & Galbraith, 2013; Stein, 2016; Wong et al., 2015). The purpose of this study was to assess sociodemographic disparities in HIL, including knowledge of health insurance terms and costs, as well as confidence in using insurance to access health care in a nationally representative sample.

## Methods

We conducted a secondary analysis of data from the Health Reform Monitoring Survey (HRMS), a cross-sectional, probability-based, nationally representative Internet survey (Holahan & Long, 2017). Although the HRMS data have been collected quarterly since 2013, the design for this sample was cross-sectional and descriptive, with all data collected within a 9-month period (third quarter of 2015 to the first quarter of 2016). The sample comprises 15,168 participants, between ages 18 and 64 years. This study met the federal criteria to quality as an exempt study by the University of Kentucky, Institutional Review Board (IRB Number 42719).

Demographic characteristics included age, sex, race and ethnicity, education, marital status, household income, working status, place of residence (metropolitan/nonmetropolitan), U.S. citizenship, and insurance coverage. Insurance coverage was categorized as private only, public only (i.e., Medicare, Medicaid), a combination of public and private, other (including Tricare and Indian Health Service), or uninsured.

HIL was measured using two multi-item, self-reported measures: (1) knowledge of terms related to health insurance and (2) confidence in the use of insurance. Participants rated items from both HIL measures on a 4-point scale: 1 (*very confident*), 2 (*somewhat confident*), 3 (*not very confident*), and 4 (*not at all confident*). For knowledge of terms, individual items asked about knowledge of premium, deductible, copayments, coinsurance, maximum annual out-of-pocket spending, provider network, and covered services. The confidence-in-use score included items such as “find a doctor or other health provider who is in your health plan's network,” “figure out whether a service is covered by your plan,” “figure out which prescription drugs are covered by your plan,” and “figure out how much a health care visit or service will cost you.” Those who responded at least *somewhat confident* (2) to knowledge and confidence questions were coded as having “adequate” knowledge or confidence. Those who responded less confidently to at least one health insurance term or activity were coded as having “inadequate” knowledge or confidence. Although the HRMS does not use a valid and reliable measure of HIL, items are derived from Paez et al.'s (2014) initial work on developing an HIL conceptual model. Scoring for these items were based on this conceptual model and guided by scoring on the Health Insurance Literacy Measure (Paez et al., 2014). Additional HIL measures included knowledge of the respondent's annual deductibles and knowledge of their out-of-pocket costs in the preceding 12 months. For each of these two measures, participants were scored as having “adequate” knowledge if they were able to provide an estimate and “inadequate” if they were unsure.

Weighted parameter estimates and corresponding 95% confidence intervals (CIs) were used for descriptive analysis. Weighted bivariate analysis was conducted using the Rao-Scott chi-square test of association, whereas comparisons adjusted for demographic factors were made using weighted logistic modeling. Outcomes of the logistic regression models included inadequate versus adequate HIL knowledge of terms and confidence in use. Analysis was performed with SAS software, version 9.4, and used an alpha level of .05.

## Results

### Knowledge of Health Insurance Terms and Confidence in Use

In a sample of 15,168 participants, more than one-half (51%) had inadequate HIL as measured by knowledge of health insurance terms, and close to one-half (48%) had inadequate HIL as measured by confidence in health insurance use. **Table 1** summarizes the weighted frequencies for demographic characteristics. Most of the sample was between ages 25 and 64 years (84.9%), non-Hispanic (62.4%), living above the federal poverty level (81.9%), living in a metropolitan area (85.5%), U.S. citizens (92.2%), and insured (90.3%). More than one-half the sample was female (50.9%), working full- or part-time (67.6%), educated past high school (59.2%), and currently married (53.1%).

**Figure 1.**
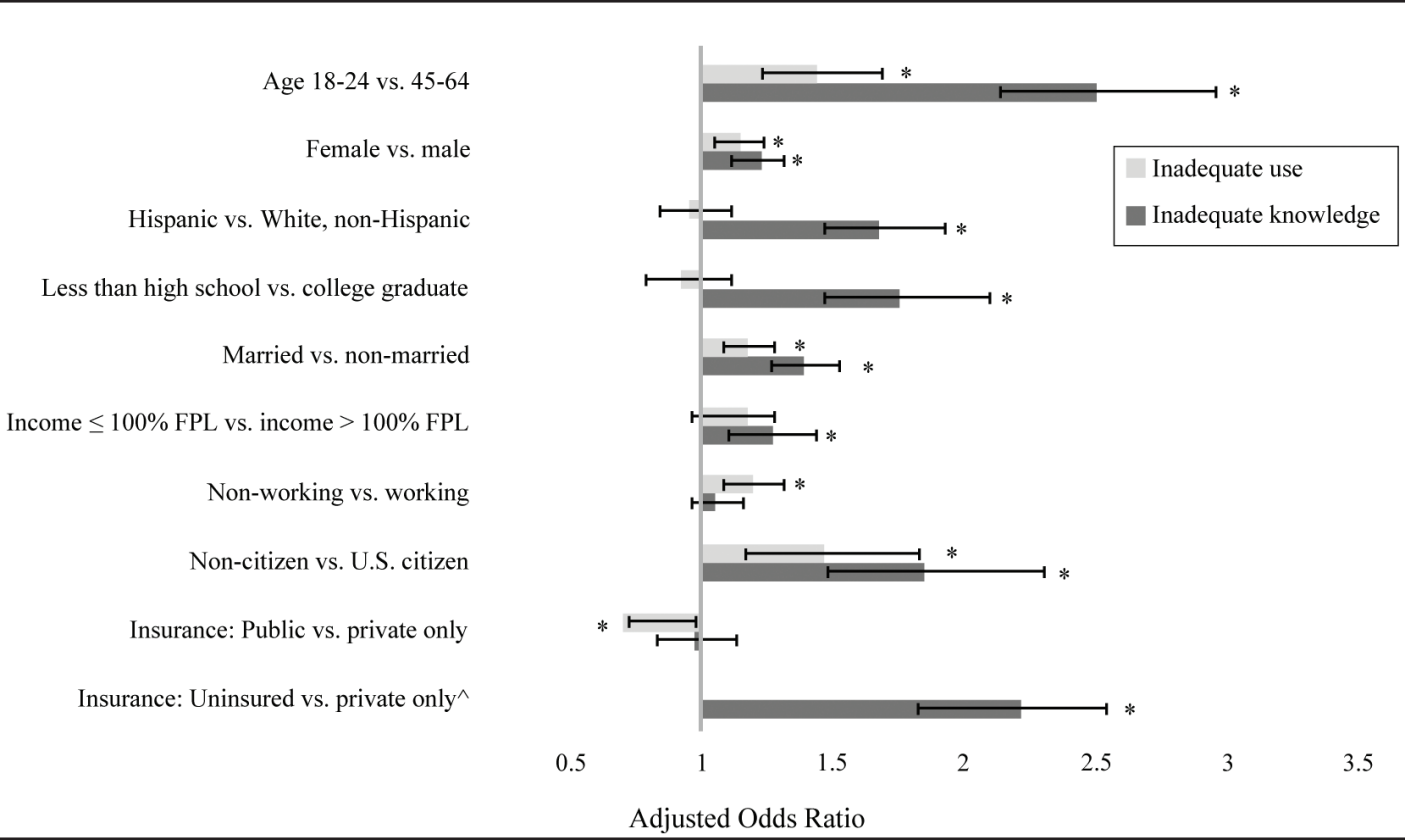
Adjusted odds ratios [95% confidence intervals] for select sociodemographic variables and inadequate health insurance literacy measured by knowledge of terms and confidence in use. FPL = federal poverty level. ^Use was not measured for uninsured. *Adjusted odds ratios with 95% confidence intervals not containing 1 are significant.

Logistic regression analysis indicated significant disparities in HIL (both in knowledge of terms and confidence in use), with the following groups at higher risk for having inadequate HIL: young adults, females, those with Hispanic ethnicity, non-U.S. citizens, and those who were currently unmarried (**Figure 1**). Also identified were people who were unemployed, had less than a high school education, had income below the federal poverty level, were uninsured, or were enrolled in public health insurance plans. For example, compared to non-Hispanic Whites, those with Hispanic ethnicity were 68% more likely to have inadequate knowledge of health insurance terms. Compared to adults between ages 44 and 64 years, young adults between ages 18 and 24 years were 151% more likely to have inadequate knowledge of terms and 44% more likely to have inadequate confidence in using health insurance. There was also a notable difference (*p* < .001) in inadequate knowledge between participants with only Medicaid coverage (57.8%) compared to those with any other type of insurance (47.6%).

As shown in **Table 2**, logistic regression was used to predict inadequate knowledge and confidence in the use of health insurance to access health care services. Findings indicated that people who were young adults, female, Hispanic, non-Hispanic Black, less educated, currently unmarried, those with incomes below the federal poverty level, and non-U.S. citizens were more likely to have inadequate knowledge of terms and inadequate confidence in use of health insurance compared to their counterparts. However, inadequate knowledge was not related to employment status or place of residence in this model. In examining inadequate confidence in using health insurance alone, the same groups as above were identified, along with those who are unemployed. Relative to those with private insurance, people with public insurance were more likely to have inadequate confidence in using health insurance.

### Knowledge of Personal Deductibles and Out-Of-Pocket Costs

In addition to examining variations in HIL across type of insurance plans, we examined the relationship between other sociodemographic factors and HIL measures, specifically those related to knowledge of health insurance deductible and out-of-pocket costs (**Table 3**). More than one-half (63.8%) of respondents were unsure about their family's out-of-pocket costs in the past year, and more than one-half (65.8%) of those with insurance coverage were unsure about their health insurance deductible amounts. For both, those with only public insurance plans had the most uncertainty, followed by private only, and a combination of public and private sources. People who were uninsured had the least uncertainty about out-of-pocket costs.

Young adults, those with less education, and those who are unemployed had a higher prevalence of having inadequate knowledge of both deductibles and out-of-pocket costs (**Table 3**). Risk of inadequate knowledge of deductibles was elevated for females and non-Hispanics Blacks (relative to Hispanics). Those living in nonmetropolitan areas were at increased risk of having inadequate knowledge of out-of-pocket costs. Those without insurance were at increased risk of not knowing out-of-pocket costs relative to those with public insurance or multiple types of insurance. Knowledge of these costs was not significantly related to income relative to the federal poverty level.

## Discussion

Findings from this study indicate low self-reported HIL nationally and significant disparities among various sociodemographic groups. More than one-half (51%) of our sample had inadequate knowledge of health insurance terms, and close to one-half (48%) had low confidence in using their insurance to access health care. According to our results, young adults, females, those with Hispanic ethnicity, non-U.S. citizens, and those who are currently unmarried, as well as those who are unemployed, have less than high school educational attainment, have income below the federal poverty level, are uninsured, or are enrolled in public health insurance plans, are more likely to have inadequate HIL. Knowledge of health insurance terms varied significantly by the respondent's main type of insurance coverage, with the lowest knowledge among the uninsured, followed by those with only public insurance coverage, those with a combination of private and public insurance, and then those with private insurance. However, there was no difference among insurance types in confidence in use, and the majority of our sample had inadequate knowledge of their family's annual out-of-pocket costs and insurance plan's deductible amounts.

These findings support the existing literature on HIL, which is itself a fairly new field of study. A more focused approach to understanding and measuring HIL began around 2011, after the passage of the Affordable Care Act (ACA) but before the enactment of its major provisions in 2014, including Medicaid expansion and insurance exchanges (Quincy, 2012). With these changes, more insurance options are available at more price points than ever before, requiring consumers, many without prior experience purchasing insurance, to make informed decisions on selecting a plan that fits their personal and financial needs (Blumberg, Long, Kenney, & Goin, 2013). Paired with continuing changes in health care reform policies, this wide range of options could lead to barriers in access for consumers with low HIL. Thus, it is important to invest in efforts to build consumer HIL skills and also to modify current health care and insurance systems to be more responsive to consumer HIL needs and abilities.

Moreover, as is supported in our results, there is great diversity among those with inadequate HIL. For example, those with limited education are often found to have inadequate levels of HIL, yet studies with college students and young adults with high educational attainment, numeracy, and general literacy have been found to have inadequate functional HIL, (Nobles, Curtis, Ngo, Vardell, & Holstege, 2019; Tilley, Yarger, & Brindis, 2018). This emphasizes the need to better address the HIL needs of young adults, especially those who are entering the workforce, transitioning from their parent's health insurance plans, and/or making independent health insurance coverage decisions.

Non-U.S. citizens are among the at-risk groups for low HIL. This group generally has less experience with the unique characteristics of the American health care system and has limited options for health insurance coverage when compared to U.S. citizens. Most immigrants are not eligible for Medicaid and have to be legally residing in the U.S. for 5 years before gaining eligibility to purchase health plans through federal and state marketplace exchanges (Centers for Medicare and Medicaid Services, 2017). Finding culturally and linguistically appropriate methods to build HIL is critical to decreasing coverage disparities, particularly among Hispanic/Latino immigrants, who have persistently higher uninsured rates even after implementation of the ACA (Doty & Collins, 2017; Edward et al., 2018).

Study findings also revealed lower levels of self-reported HIL among Medicaid recipients when compared to those enrolled in any other health insurance programs (public or private), providing some implications for state health care reform policies related to Medicaid. Prior to 2016, several states had adopted Medicaid expansion under the ACA or Section 1115 Medicaid waiver programs. Medicaid waiver programs allowed for state innovation in Medicaid administration via demonstration projects, often resulting in additional requirements for participants to maintain coverage (Rosenbaum & Hurt, 2014). Many states, including Kentucky, Arkansas, New Hampshire, and Indiana, have received approval from the Centers for Medicare and Medicaid services to incorporate work or community engagement requirements for “able bodied” Medicaid beneficiaries (Neale, 2018). Arkansas was the first state to implement a work requirement waiver program, and as of December 2018, an estimated 18,000 have lost coverage due to noncompliance with reporting requirements (Rudowitz, Musumeci & Hall, 2019). Despite the stated intent of these waivers to help promote HIL among low-income beneficiaries by requiring them to engage with the health care system, the imposition of major barriers to enrollment (including penalties and lock-out periods) on this low-HIL group may pose significant barriers health care access.

## Study Limitations

Our study has several limitations. The HIL measure used in the HRMS has not been psychometrically tested and validated in prior research and only uses subjective, self-reported measures of HIL knowledge and confidence in use, leading to cautious interpretation of findings. Our cross-sectional design and lack of longitudinal data does not allow us to fully understand the nature of the relationship between the health insurance coverage status and HIL levels of survey respondents over time. Additionally, health reform policies are implemented differently across states, especially state Medicaid policies. These variations are not adequately accounted for in this study as census tract and/or state-level data were not available through the HRMS database. Health reform policies are in a constant state of flux, making it imperative to gather state-level data that can help us better understand the impact of the changing health care climate on population health outcomes.

## Policy Implications

Our findings have significant and immediate policy implications, especially in terms of empowering consumers with the knowledge and resources to support informed health insurance decision-making. Recent changes to key components of the ACA, including the elimination of penalties associated with the individual mandate, and imposition of Medicaid 1115 waiver requirements noted above, will profoundly affect the knowledge level and skills consumers will need to select, obtain, use, and maintain health insurance coverage (Carroll, 2018; Jost, 2018). This may serve as a challenge for many as findings from this study and others (Paez et al., 2014; Long et al., 2014) show that the majority of U.S. population does not understand basic health insurance terms, but that people with higher HIL are more likely to be insured (Hoerl et al., 2017). Supporting consumer decision-making with a focus on improving HIL could be essential to improving health insurance coverage and understanding access, cost, and quality trends in a competitive health care market (Barnes, Hanoch & Rice, 2015). Despite this limitation, changes in health reform policies continue to place higher levels of personal responsibility on consumers to independently make informed decisions about their health (Long et al. 2014). Without adequate levels of HIL, consumers are unable to understand the financial and health implications of health insurance plans, or how to successfully use their own plan when accessing needed care (Kim, Braun & Williams, 2013; Brown et al., 2016).

## Conclusions

With more than one-half the U.S. population reporting low HIL, increased efforts are needed in promoting health insurance knowledge and supporting informed health care decision-making in socioeconomically diverse populations. Given the drastic changes in health reform policies at federal and state levels after the implementation of the ACA, it is imperative to identify and strengthen consumers' abilities to select health insurance, use it appropriately, and adapt when their life circumstances change across their lifespan (e.g., aging out of parental plans, changing jobs, becoming unemployed, or retiring). Changes also need to be made in the health system, especially in promoting value-based care and supporting delivery system reforms. Health care and health insurance industries need to design or redesign their services and materials to be more responsive to consumer HIL needs and abilities. Future research is needed to develop and test effective methods to improve consumer HIL and decrease associated health disparities.

## Figures and Tables

**Table 1:** Summary of Sociodemographic Characteristics of the Study Sample (*N* = 15,168)

**Characteristic**	***n***	**Weighted % [95% CI]**

Age		
18–24 years	1,280	15.1 [14.3, 15.9]
25–44 years	5,922	42.2 [41.3, 43.1]
45–64 years	7,966	42.7 [41.8, 43.5]

Sex		
Male	7,592	49.1 [48.2, 50]
Female	7,576	50.9 [50, 51.8]

Race/ethnicity^a^		
White, non-Hispanic	10,460	62.4 [61.5, 63.3]
Hispanic	2,258	17.1 [16.4, 17.8]
Black, non-Hispanic	1,485	12.3 [11.6, 12.9]
Other race, non-Hispanic	551	6.9 [6.3, 7.45]
More than one race, non-Hispanic	414	1.4 [1.2, 1.5]

Education		
Less than high school	1,382	11.4 [10.7, 12]
High school diploma/GED	4,312	28.5 [27.7, 29.3]
Some college	4,417	28.9 [29, 30.7]
College graduate	5,057	30.3 [29.5, 31.1]

Married		
Yes	8,620	53.1 [52.2, 54]
No	6,548	46.9 [46, 47.8]

Family Income ≥100% of FPL^a^		
Yes	12,572	81.9 [81.2, 82.7]
No	2,251	18.1 [17.3, 18.8]

Working full- or part-time		
Yes	10,363	67.6 [66.8, 68.5]
No	4,805	14.5 [13.9, 15.1]

Place of residence		
Metropolitan	13,050	85.5 [84.9, 86.1]
Nonmetropolitan	2,118	14.5 [13.9, 15.1]

U.S. citizen^a^		
Yes	13,947	92.2 [91.6, 92.7]
No	867	7.8 [7.3, 8.4]

Time of survey		
Quarter 3 of 2015	7,648	50.4 [49.5, 51.3]
Quarter 1 of 2016	7,520	49.6 [48.7, 50.5]

Type of coverage in past 12 months^b^		
From current/former employer	8,897	62 [61.1, 62.9]
Medicaid	2,173	18 [17.3, 18.8]
From insurance company	1,893	13.7 [13.1, 14.4]
Medicare	1,180	7.7 [7.2, 8.2]
Other	871	6.9 [6.4, 7.4]
Tricare	784	5.9 [5.4, 6.3]
Indian health services	221	2.4 [1.8, 2.4]

Note. CI = confidence interval; FPL = federal poverty level; GED = General Education Diploma.

a Total sum may not equal to 15,168 due to missing data or refusal of participant to answer.

b Weighted percentages for types of insurance sum to more than 100 because some people have more than one source of insurance coverage.

**Table 2:** Adjusted Logistic Regressions Modeling Inadequate Knowledge of Insurance Terms and Confidence in Use of Health Insurance

**Characteristic**	**Inadequate Knowledge of Terms (*n* = 13,994)**			**Inadequate Confidence in Use (*n* = 12,816)**		

	**Adjusted OR**	**95% CI for OR**	***p*^a^**	**Adjusted OR**	**95% CI for OR**	***p*^b^**

Age						
18–24 years	2.51	[2.14, 2.95]	<.001	1.44	[1.23, 1.68]	<.001
25–44 years	1.43	[1.32, 1.55]	<.001	1.12	[1.03, 1.22]	<.001
45–64 years	ref	-	-	ref	-	-

Sex						
Female	1.21	[1.12, 1.31]	<.001	1.14	[1.05, 1.23]	.007
Male	ref	-	-	ref	-	-

Race/ethnicity						
Hispanic	1.68	[1.47, 1.92]	<.001	0.97	[0.85, 1.11]	.70
Other race, non-Hispanic	1.50	[1.22, 1.84]	<.001	1.31	[1.07, 1.61]	.008
Black, non-Hispanic	0.97	[0.85, 1.11]	.64	0.71	[0.61, 0.81]	<.001
More than one race, non-Hispanic	0.97	[0.75, 1.25]	.79	1.07	[0.84, 1.37]	.57
White, non-Hispanic	ref	-	-	ref	-	-

Education						
Less than high school	1.75	[1.47, 2.09]	<.001	0.94	[0.79, 1.12]	.48
High school diploma/GED	1.18	[1.07, 1.31]	.001	0.83	[0.75, 0.93]	<.001
Some college	0.99	[0.90, 1.10]	.92	0.78	[0.71, 0.86]	<.001
College graduate	ref	-	-	ref	-	-

Married						
Yes	ref	-	-	ref	-	-
No	1.39	[1.27, 1.52]	<.001	1.17	[1.08, 1.28]	<.001

Family income <100% of FPL						
Yes	1.26	[1.10, 1.44]	<.001	1.10	[0.96, 1.28]	.18
No	ref	-	-	ref	-	-

Working full- or part-time						
Yes	ref	-	-	ref	-	-
No	1.06	[0.96, 1.16]	.27	1.19	[1.08, 1.31]	<.001

Place of residence						
Metropolitan	ref	-	-	ref	-	-
Nonmetropolitan	1.03	[0.92, 1.16]	.62	1.10	[0.98, 1.24]	.56

U.S. citizen						
Yes	ref	-	-	ref	-	-
No	1.85	[1.48, 2.30]	<.001	1.46	[1.17, 1.82]	<.001

Insurance coverage						
Private	ref	-	-	ref	-	-
Public	0.98	[0.84, 1.13]	.76	0.85	[0.73, 0.98]	.026
Combination or other	1.04	[0.92, 1.18]	.53	0.89	[0.79, 1.01]	.067
Uninsured	2.15	1.82, 2.53]	<.001	-	-	-

Note. CI = confidence interval; FPL = federal poverty level; GED = General Education Diploma; OR = odds ratio; ref = reference.

a *p* from adjusted logistic regression (*n* = 13,994) modeling inadequate knowledge, adjusted for all other variables in the table and cohort (Quarter 3 2015 or Quarter 1 2016). There was no difference between cohorts for this outcome. Only respondents with complete data on all variables in the models included in the regression.

b *p* from adjusted logistic regression (*n* = 12,816) modeling inadequate confidence in use among those with insurance (*n* =13,901), adjusted for all other variables in the table and cohort (Quarter 3 2015 or Quarter 1 2016). There was no difference between cohorts for this outcome. Only respondents with complete data on all variables in the models included in the regression.

**Table 3:** Adjusted Logistic Regressions Modeling Inadequate Knowledge of Health Care Deductibles and Out-of-Pocket Costs

**Characteristic**	**Inadequate Knowledge of Annual Deductible per Person Under Health Plan (*n* = 12,816)**			**Inadequate Knowledge of Last 12-month Out-of-Pocket Health Care Costs for You and Your Family (*n* = 13,994)**		

	**Adjusted OR**	**95% CI for OR**	***p*^a^**	**Adjusted OR**	**95% CI for OR**	***p*^b^**

Age						
18–24 years	2.36	[2.02, 2.76]	<.001	2.62	[2.26, 3.05]	<.001
25–44 years	1.26	[1.15, 1.38]	<.001	1.22	[1.12, 1.34]	<.001
45–64 years	ref	-	-	ref	-	-

Sex						
Female	1.02	[0.94, 1.11]	.65	1.11	[1.01, 1.21]	.017
Male	ref	-	-	ref	-	-

Race/ethnicity						
Other race, non-Hispanic	1.61	[1.31, 1.99]	<.001	1.17	[0.94, 1.46]	.17
Black, non-Hispanic	1.33	[1.15, 1.54]	<.001	1.36	[1.19, 1.57]	<.001
Hispanic	1.26	[1.09, 1.46]	.002	1.09	[0.95, 1.26]	.22
More than one race, non-Hispanic	1.08	[0.83, 1.41]	.58	0.93	[0.71, 1.21]	.58
White, non-Hispanic	ref	-	-	ref	-	-

Education						
Less than high school	1.76	[1.44, 2.13]	<.001	1.88	[1.57, 2.25]	<.001
High school diploma/GED	1.57	[1.40, 1.76]	<.001	1.64	[1.46, 1.84]	<.001
Some college	1.36	[1.22, 1.52]	<.001	1.49	[1.33, 1.66]	<.002
College graduate	ref	-	-	ref	-	-

Married						
Yes	ref	-	-	ref	-	-
No	1.35	[1.23, 1.49]	<.001	1.07	[0.98, 1.18]	.14

Family income <100% of FPL						
Yes	0.87	[0.75, 1.02]	.096	0.96	[0.83, 1.10]	.55
No	ref	-	-	ref	-	-

Working full- or part-time						
Yes	ref	-	-	ref	-	-
No	1.15	[1.03, 1.28]	.014	1.21	[1.09, 1.34]	<.001

Place of residence						
Metropolitan	ref	-	-	ref	-	-
Nonmetropolitan	0.98	[0.87, 1.12]	.81	1.16	[1.03, 1.31]	.015

US citizen						
Yes	ref	-	-	ref	-	-
No	1.32	[1.05, 1.65]	.018	1.13	[0.92, 1.38]	.26

Insurance coverage						
Private	ref	-	-	ref	-	-
Public	0.55	[0.47, 0.65]	<.001	0.62	[0.52, 0.72]	<.001
Combination or other	0.98	[0.86, 1.11]	.72	0.92	[0.81, 1.05]	.23
Uninsured	-	-	-	1.17	[0.99, 1.39]	.057

Note. CI = confidence interval; FPL = federal poverty level; GED = General Education Diploma; OR = odds ratio; ref = reference.

a *p* from adjusted logistic regression (*n* = 12,816) modeling inadequate knowledge of deductibles among those with insurance (*n* =13,901), adjusted for all other variables in the table and cohort (Quarter 3 2015 or Quarter 1 2016).

b *p* from adjusted logistic regression (*n* = 13,994) modeling inadequate knowledge of out-of-pocket costs, adjusted for all other variables in the table and cohort (Quarter 3 2015 or Quarter 1 2016). There was no difference between cohorts for this outcome.
